# Maternal Antiviral Immunoglobulin Accumulates in Neural Tissue of Neonates To Prevent HSV Neurological Disease

**DOI:** 10.1128/mBio.00678-17

**Published:** 2017-07-05

**Authors:** Yike Jiang, Chaya D. Patel, Richard Manivanh, Brian North, Iara M. Backes, David A. Posner, Francesca Gilli, Andrew R. Pachner, Lananh N. Nguyen, David A. Leib

**Affiliations:** a Department of Microbiology and Immunology, Geisel School of Medicine at Dartmouth, Lebanon, New Hampshire, USA; b Department of Neurology, Geisel School of Medicine at Dartmouth, Lebanon, New Hampshire, USA; c Department of Pathology, Geisel School of Medicine at Dartmouth, Lebanon, New Hampshire, USA; Columbia University Medical College

**Keywords:** herpes simplex virus, maternal antibody, neonatal infection, neuroimmunology, trigeminal ganglion

## Abstract

While antibody responses to neurovirulent pathogens are critical for clearance, the extent to which antibodies access the nervous system to ameliorate infection is poorly understood. In this study on herpes simplex virus 1 (HSV-1), we demonstrate that HSV-specific antibodies are present during HSV-1 latency in the nervous systems of both mice and humans. We show that antibody-secreting cells entered the trigeminal ganglion (TG), a key site of HSV infection, and persisted long after the establishment of latent infection. We also demonstrate the ability of passively administered IgG to enter the TG independently of infection, showing that the naive TG is accessible to antibodies. The translational implication of this finding is that human fetal neural tissue could contain HSV-specific maternally derived antibodies. Exploring this possibility, we observed HSV-specific IgG in HSV DNA-negative human fetal TG, suggesting passive transfer of maternal immunity into the prenatal nervous system. To further investigate the role of maternal antibodies in the neonatal nervous system, we established a murine model to demonstrate that maternal IgG can access and persist in neonatal TG. This maternal antibody not only prevented disseminated infection but also completely protected the neonate from neurological disease and death following HSV challenge. Maternal antibodies therefore have a potent protective role in the neonatal nervous system against HSV infection. These findings strongly support the concept that prevention of prenatal and neonatal neurotropic infections can be achieved through maternal immunization.

## IMPORTANCE

Herpes simplex virus 1 is a common infection of the nervous system that causes devastating neonatal disease. Using mouse and human tissue, we discovered that antiviral antibodies accumulate in neural tissue after HSV-1 infection in adults. Similarly, these antibodies pass to the offspring during pregnancy. We found that antiviral maternal antibodies can readily access neural tissue of the fetus and neonate. These maternal antibodies then protect neonatal mice against HSV-1 neurological infection and death. These results underscore the previously unappreciated role of maternal antibodies in protecting fetal and newborn nervous systems against infection. These data suggest that maternal immunization would be efficacious at preventing fetal/neonatal neurological infections.

## INTRODUCTION

The nervous system was historically considered immune privileged based on graft tolerance and specialized barrier structures, but it is now clear that immune privilege is neither homogenous nor absolute (1, 2). Immune responses vary depending on the specific neural compartment, and immune privilege can be chronically altered by infection (3). In fact, human neural tissue is seldom sterile and harbors lifelong pathogens such as *Toxoplasma gondii*, varicella-zoster virus, and human herpesvirus 6 (4–8). The most common neurotropic pathogen (infecting ~90% of humans) is herpes simplex virus (HSV), which is known to alter the immune microenvironment within the nervous system (9–11). HSV-1 infects and establishes latency in neurons, especially those of the trigeminal ganglia (TG), wherein immune cells and cytokines persist long after the primary infection has been cleared (12–14). Although B cells are clearly crucial for viral resistance (15, 16), humoral immunity against HSV-1 in neural tissue remains poorly understood.

Antibody access to the nervous system is especially relevant to vertically transmitted infections. The TORCH pathogens (*Toxoplasma*, other, rubella, cytomegalovirus, and HSV) and Zika virus are known to cause neurological sequelae in fetuses and neonates (17, 18). Though clearly vulnerable to infection, this population attains some measure of protection from maternal antigen-specific IgG. However, whether these maternal antibodies directly protect the fetus or neonate against neurological infections remains unknown. Neonatal HSV is a devastating disease that can result in serious long-term sequelae and even death in newborns (19). Based on clinical observations, it has been hypothesized that maternal antibodies protect the neonate from neurotropic HSV (20, 21). The risk of transmission from latently infected mothers is less than 1%, despite recurrent HSV shedding (20, 22). In contrast, transmission risk to the baby can be as high as 50% during parturition from mothers with newly acquired genital HSV infections (20, 23). Although there is general support for the idea that maternal antibody may protect the neonatal nervous system from HSV (21, 22, 24–26), this has not been directly tested or therapeutically translated.

In this study, we evaluated naive and HSV-infected neural tissue of mice and humans for the presence of antibody. We demonstrate the long-term presence of HSV-specific IgG in both mice and human TG, long after the clearance of replicating virus. Furthermore, our data show that IgG was not only made within the infected TG, but it also permeated from systemic circulation into infected and naive TG. This led us to the discovery that maternal antibodies can similarly transfer into naive fetal and neonatal TG, protecting the neonate against neurological HSV-1 spread and mortality. Taken together, this work highlights the importance of maternal antibodies in protecting the newborn nervous system from viral infections. These findings strongly support the concept that prevention of prenatal and neonatal neurotropic infections can be achieved through maternal immunization.

## RESULTS

### Persistence of HSV-specific IgG in the TG during latency.

**FIG 1:**
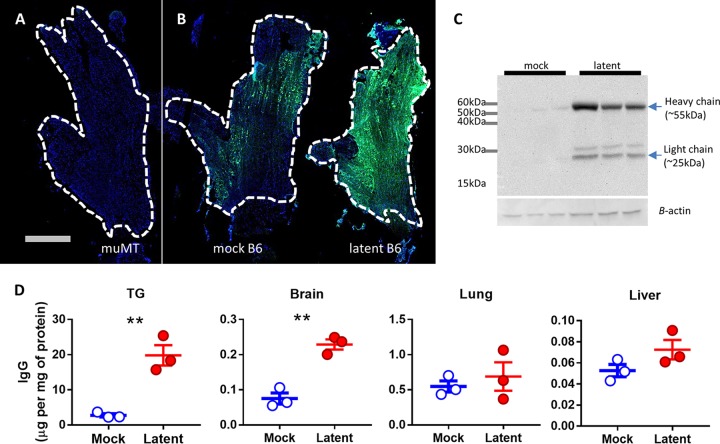
Antibodies persist in the TG during HSV-1 latency. Mice were infected with 2 × 10^6^ PFU of HSV-1 via the ocular route, and TG were harvested following the establishment of latency. Panels A and B show representative stitched IF images of entire TG stained with 4′,6-diamidino-2-phenylindole (DAPI) (blue) and anti-mouse IgG (green): (A) latently infected muMT (background control); (B) mock-infected B6 and latently infected B6. The scale bar represents 1 mm. Dashed white lines outline the margins of the TG. (C) Western blot of TG protein homogenates probed for mouse IgG. Each lane is loaded with protein from a pair of TG from one mouse. (D) ELISA of IgG protein level normalized to total protein concentration of organ homogenate. Error bars represent the standard error of the mean (SEM). Statistical significance was determined by unpaired parametric *t* test. **, *P* < 0.005. Data represent 2 to 4 independent experiments.

The persistence of myeloid cells and T cells in the TG during latent HSV-1 infection is well documented (10, 13). However, we wanted to address if this also applied to humoral immunity. Upon ocular infection, HSV replicates in the cornea and spreads in a retrograde direction along the ciliary nerves into the TG, where it establishes latency (27, 28). In this study, we harvested perfused latently infected TG at ≥30 days postinfection (dpi) (29). Using immunofluorescence (IF), we found generalized and elevated staining for IgG in latently infected TG relative to mock-infected TG (Fig. 1A and B). There was no change in IgG from baseline levels at 7 dpi, but robust increases were evident at 20 dpi and persisted out to 8 months after infection, the latest time point taken (see Fig. S1 in the supplemental material). To characterize the IgG response further, we visualized the IgG heavy (55-kDa) and light (25-kDa) chains in TG by Western blotting (Fig. 1C). Compared to the faint IgG signals in the mock-infected TG, latently infected TG showed a strong increase in IgG signals, consistent with the IF data (Fig. 1B). We next quantified the amount of IgG relative to total tissue protein using an enzyme-linked immunosorbent assay (ELISA) and bicinchoninic acid (BCA) assay (Fig. 1D). Compared to tissue from mock-infected animals, we found that IgG concentrations were significantly increased in the TG (*P* = 0.004) and brain (*P* = 0.002) but not in the lung (*P* = 0.55) or liver (*P* = 0.14). The magnitude of change, however, was much greater in the TG than in other tissues. Taken together, these data suggest that there is a long-term persistence of IgG in neural tissue after HSV-1 infection.

10.1128/mBio.00678-17.2 FIG S1  Increased antibody presence in the TG during HSV-1 latency. Shown is IF of TG sections stained with DAPI (blue), anti-IgG (green), and anti-CD45 (violet) from mice corneally infected with 2 × 10^5^ PFU HSV-1. Stitched images are presented. (A) TG of mock-infected mice as the background control. (B to E) TG were perfused and harvested at the indicated time points after infection. Data represent 2 to 3 similar experiments. Download FIG S1, TIF file, 2.6 MB. Copyright © 2017 Jiang et al. 2017 Jiang et al. This content is distributed under the terms of the Creative Commons Attribution 4.0 International license.

**FIG 2:**
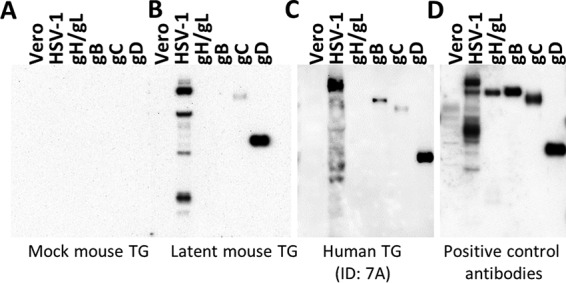
IgG in the TG binds to HSV-1 antigens. Shown are Western blots loaded with 10 ng of protein: mock viral stock lysate (Vero), HSV-1 viral stock lysate (HSV-1), purified gH/L, gB, gC, and gD. These blots were probed with TG homogenates made from (A) mock-infected mice, (B) latently infected mice, or (C) human autopsy samples (ID no. 7A, as referenced in Table S1). (D) Blot probed with a pool of monoclonal antibodies against individual glycoproteins as a positive control. Data represent 3 to 5 independent experiments. See Fig. S2 for additional blots.

To address whether the TG IgGs were specific for HSV-1, we used a modified Western blot. In this procedure, lysate of uninfected Vero cells, lysate of HSV-1 grown in Vero cells, and purified viral glycoproteins were run on Western blots. Homogenates of TG from mock-infected mice, latently infected mice, and human autopsies were then used to probe the blots as primary antibodies (Fig. 2). TG IgGs bound to protein bands were then visualized using anti-mouse or anti-human IgG secondary antibodies. Mock-infected TG homogenates failed to recognize Vero cell or HSV proteins (Fig. 2A), while latently infected murine TG contained IgG that bound to multiple proteins in HSV-infected Vero cells (Fig. 2B). We found no evidence for IgG binding to uninfected Vero cell lysates. Extracts from latently infected TG recognized various permutations of gB, gC, and gD but not gH/gL (Fig. 2B; see Fig. S2A in the supplemental material). Because the purified recombinant glycoproteins are truncated proteins, their sizes do not match specific bands in the HSV-infected Vero cell lysates (30). We next addressed whether such HSV-specific antibodies also existed in human TG. In all autopsy-derived human TG that screened positively for the HSV-1 genome (see Table S1 in the supplemental material), we found anti-HSV IgG with a binding pattern that resembled the mouse model, with variable reactivity to gB, gC, and gD (Fig. 2C; Fig. S2B). Intriguingly, we also observed reactivity to the immediate early protein ICP4 (Fig. S2C). Taken together, these data demonstrate the long-term presence of HSV-specific IgG in murine and human TG.

10.1128/mBio.00678-17.3 FIG S2  Western blots showing IgG in mouse and human TG binding to HSV-1 antigens. Panels A and B show Western blots loaded with 50 ng of mock-infected Vero cell lysate, HSV-1 stock lysate, and purified gH/L, gB, gC, and gD. These blots were probed with TG homogenates made from (A) mouse samples and (B) human samples (ID no. 1A and 2A as referenced in Table S1). (C) Western blot loaded with 50 ng of purified ICP4, ICP8, ICP27, U_L_30, U_L_42, and lysates from uninfected Dick-1 cells, Dick-1 cells infected with HSV-1 for 6 h, uninfected Vero cells, and Vero cells infected with HSV-1. The blot was probed with TG homogenate made from human samples. Download FIG S2, TIF file, 1.3 MB. Copyright © 2017 Jiang et al. 2017 Jiang et al. This content is distributed under the terms of the Creative Commons Attribution 4.0 International license.

10.1128/mBio.00678-17.6 TABLE S1  Summary of adult human TG-IgG binding profiles. Human TG samples were obtained from autopsies. Each patient is denoted by a number, and individual TG in the pair are denoted by A and B. TG from the same individual (i.e., 2A and 2B) were processed and tested separately. The HSV-1 genome was assayed by PCR for HSV-1 gD. Modified Western blotting was performed on human samples as shown in Fig. 2D, and the IgG binding pattern to HSV antigens is summarized. Download TABLE S1, TIF file, 0.2 MB. Copyright © 2017 Jiang et al. 2017 Jiang et al. This content is distributed under the terms of the Creative Commons Attribution 4.0 International license.

### TG IgG originates both from local sources and from circulation.

**FIG 3:**
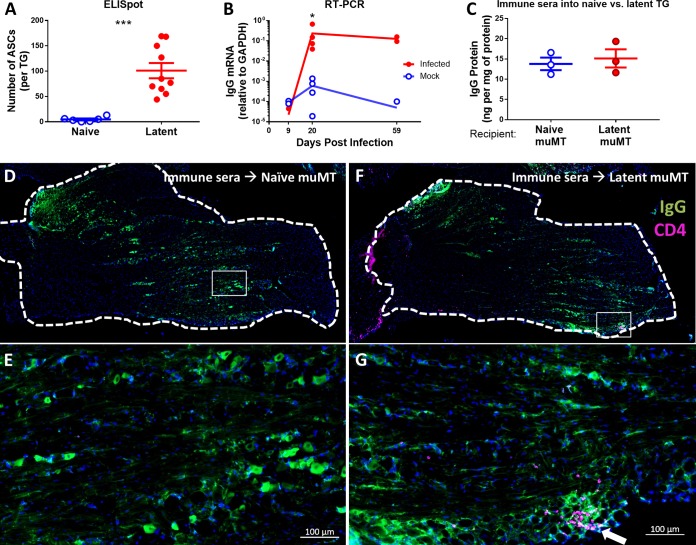
TG IgG originates both locally and from circulation. For panels A and B, B6 mice were latently infected via the ocular route with 1 × 10^5^ PFU HSV-1. (A) Number of antibody-secreting cells in TG cell suspensions as measured by ELISpot. (B) Expression levels of IgG heavy chain relative to GAPDH in TG as measured by RT-PCR. For panels C through G, 100 µl of HSV-1 immune serum was injected intraperitoneally into recipient naive or latently infected muMT mice, and after 18 h, perfused TG were harvested. (C) Concentration of injected IgG in recipient TG normalized to protein concentration as determined by ELISA and BCA assay. For panels D through G, perfused TG were stained with DAPI (blue), anti-IgG (green), and anti-CD4 (violet). (D) IF of TG from recipient naive muMT mouse. (E) Enlarged image of boxed area from panel D. (F) IF of TG from recipient latently infected muMT mouse. (G) Enlarged image of boxed area in panel F. The arrow points to a cluster of CD4^+^ cells. Scale bars represent 100 µm. Statistical significance was determined by Mann-Whitney test (A and B) or unpaired parametric *t* test (C). *, *P* < 0.05; ***, *P* < 0.001. Data represent 2 to 3 independent experiments.

Having shown the existence of HSV-specific TG IgG, we wished to assess whether it was synthesized and secreted locally within the TG or made elsewhere and subsequently localized to the TG. Based on our observation of IgG^+^ CD45^+^ cells in latently infected TG by IF (Fig. S1), we hypothesized that there was local IgG production within the TG. We performed enzyme-linked immunospot (ELISpot) analysis to examine for antibody-secreting cells (ASCs) and measured IgG mRNA by reverse transcription-PCR (RT-PCR). Consistent with the hypothesis of local IgG production, we observed an average of ~100 ASCs per latently infected TG compared to background levels (<10 spots) in naive TG (Fig. 3A). Furthermore, while there was no change in expression of IgG mRNA in mock-infected and infected TG at 9 dpi, we observed significantly elevated expression in the infected TG at 20 and 59 dpi relative to controls (Fig. 3B). Although the identity of these ASCs is currently under study, these data suggest that the increase in TG IgG during latency is likely maintained by local ASCs.

The presence of local ASCs in latently infected TG, however, did not exclude the possibility that circulating IgG could translocate to HSV-infected TG. To test this idea, we harvested sera from latently infected mice (immune sera), transferred the sera into naive or latently infected muMT mice, and examined their TG using IF and ELISA. Although muMT mice have some IgM-independent IgG production (31, 32), we found no evidence of IgG in muMT TG by IF or ELISA. IgG detected in the TG in this experiment must therefore be derived from injected C57BL/6 (B6) mouse immune sera. Unexpectedly, ELISA showed equivalent IgG levels in latently infected and naive TG (Fig. 3C). This was further supported by IF data demonstrating comparable staining for IgG in both naive (Fig. 3D and E) and latently infected (Fig. 3F and G) TG. It was recently shown that infiltrating CD4^+^ T cells mediate IgG access to neural tissue (33), and in accordance, we observed CD4^+^ T cells in latently infected TG (Fig. 3G; white arrow). The equivalence, however, of IgG levels in naive and latently infected TG suggests that CD4^+^ T cells were not absolutely required for this IgG entry. To rule out the possibility that IgG was restricted to the TG vasculature, we visualized blood vessels by IF and observed that the IgG signal was nonvascular and localized to the neural parenchyma (see Fig. S3 in the supplemental material). Taken together, the upregulation of TG IgG during viral latency (Fig. 1) is most likely due to local IgG production by persisting ASCs that are recruited during HSV-1 infection and not due to greater permeability to circulating IgG. However, these data also demonstrate that circulating IgG can access naive TG independently of infection and inflammation.

10.1128/mBio.00678-17.4 FIG S3  Passively administered IgG accesses extravascular TG tissue. Immune sera were injected into naive or latently infected muMT mice, and perfused TG were harvested at 18 h as in Fig. 3. (A to C) Representative IF images of TG from (A) uninjected naive muMT (negative control), (B) injected naive muMT, and (C) injected latently infected muMT mice that were stained for IgG (green), CD31/PECAM-1 (red), and DAPI (blue). Scale bars represent 100 μm. Download FIG S3, TIF file, 2.2 MB. Copyright © 2017 Jiang et al. 2017 Jiang et al. This content is distributed under the terms of the Creative Commons Attribution 4.0 International license.

### Maternal antiviral IgG accesses fetal and neonatal TG.

**FIG 4:**
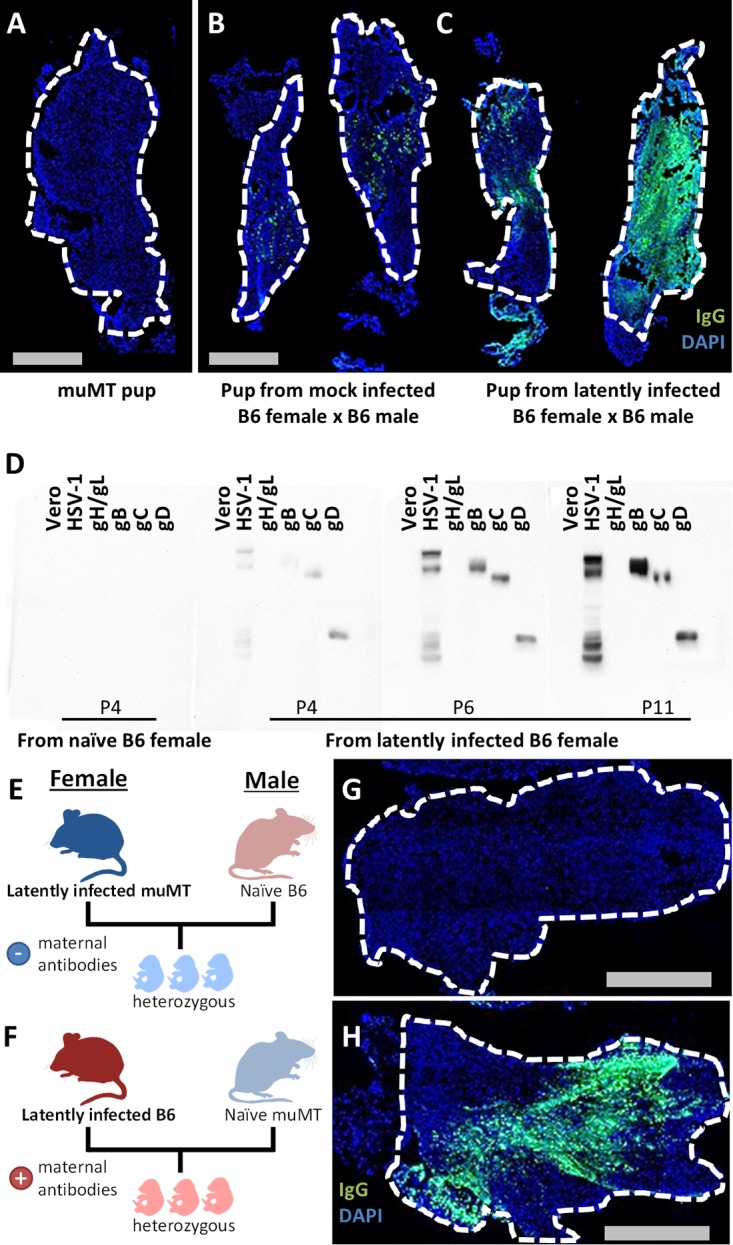
Maternal IgG transfers into naive newborn mouse TG. For IF images, neonatal TG were stained with anti-IgG (green) and DAPI (blue). (A) IF of P9 muMT TG as background control. (B and C) IF of TG pairs from P6 pups born to (B) mock-infected B6 female and (C) latently infected B6 female, both of which were bred to naive B6 males. (D) Western blot of indicated samples probed with neonatal TG homogenates from pups born to naive or latently infected B6 females at the indicated ages. (E and F) Breeding scheme to show the generation of heterozygous pups from muMT mated to B6 breeders. Latently infected muMT female bred to naive B6 male results in pups without maternal antibodies (E). Latently infected B6 female bred to naive muMT male results in pups with maternal antibodies (F). (G) IF of P15 TG corresponding to the breeding scheme in panel E. (H) IF of P15 TG corresponding to the breeding scheme in panel F. Data represent 2 independent experiments.

The observation that circulating IgG could access naive TG implies that there would be analogous transfer of maternal IgG into naive fetal or neonatal neural tissue. This line of reasoning is timely because of renewed interest in preventing neurological sequelae of Zika and the TORCH pathogens (17, 34). Using IF and Western blotting, we examined TG of newborn mice from mock-infected and latently infected C57BL/6 (B6) females bred to naive B6 males. Using the TG of purebred muMT pups as the background control (Fig. 4A), we observed the presence of TG IgG in pups regardless of maternal HSV infection status (Fig. 4B and C). That said, levels of IgG appeared higher in TG of neonates born to latently infected females, and only neonates from latently infected females had TG IgGs that were specific for HSV (Fig. 4D). The apparent accumulation of anti-HSV IgG from postnatal day 4 (P4) to P11 is most likely due to the continual entry and retention of circulating IgG in neonatal TG, which are visibly growing postpartum. To prove that these antibodies were maternal in origin, latently infected muMT and B6 females were bred to naive males of the opposite genotype (latently infected muMT female × naive B6 male and latently infected B6 female × naive muMT male [Fig. 4E and F]). All F1 mice were therefore genotypically comparable but born to latently infected females that differed in whether they could make antibodies. TG of pups from latently infected muMT females showed no IgG staining (Fig. 4G), while TG of pups from latently infected B6 females showed robust staining for IgG (Fig. 4H). This experiment demonstrated that the TG IgG in the neonate is exclusively maternal.

**FIG 5:**
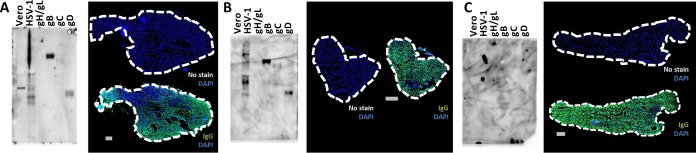
Naive human fetal TG harbor anti-HSV IgG. TG pairs from three human fetuses (A, B, and C) were assayed by modified Western blotting (left) and IF (right). The gestational ages were (A) 15 weeks, (B) 22 weeks, and (C) 21 weeks. Modified Western blots using human fetal TG homogenates were performed to assess for anti-HSV IgG. The blot was overexposed in panel C in an attempt to show reactive bands. Serial sections of the contralateral fetal TG were stained with DAPI (blue) and anti-human IgG (green) or no secondary antibody (no stain) as the background control. The scale bar represents 1 mm. All TG were HSV-1 DNA negative. See Fig. S3 for HSV-1 genome PCRs.

To address the translational relevance of these results, we assessed whether maternal IgG could also transfer into naive TG in humans. To do this, we evaluated three pairs of human fetal TG, all of which were confirmed negative for the HSV genome (see Fig. S4 in the supplemental material). By IF, all three samples were positive for human IgG (Fig. 5). More specifically, two samples were positive for anti-HSV IgG (Fig. 5A and B) and one was negative (Fig. 5C). While this sample size is small, it is intriguing that this 2:1 ratio corresponds with the ~60% of the female population of childbearing age that is infected with HSV-1 (35). Taking the human and mouse data together, we concluded that maternal IgG readily transfers and persists in naive fetal and neonatal TG.

10.1128/mBio.00678-17.5 FIG S4  PCR for HSV-1 genome in human fetal TG. DNA was extracted from human fetal TG, and PCR was performed. (A) PCR for glycoprotein D with the expected band of 220 bp. (B) PCR for RNase P with the expected band of 80 bp. Controls: +, HSV genomes mixed with HEK293T DNA; −, HEK293T DNA only; =, H_2_O control. Download FIG S4, TIF file, 0.4 MB. Copyright © 2017 Jiang et al. 2017 Jiang et al. This content is distributed under the terms of the Creative Commons Attribution 4.0 International license.

### Maternal antibodies protect neonates from HSV-1 intranasal infection and disseminated disease.

**FIG 6:**
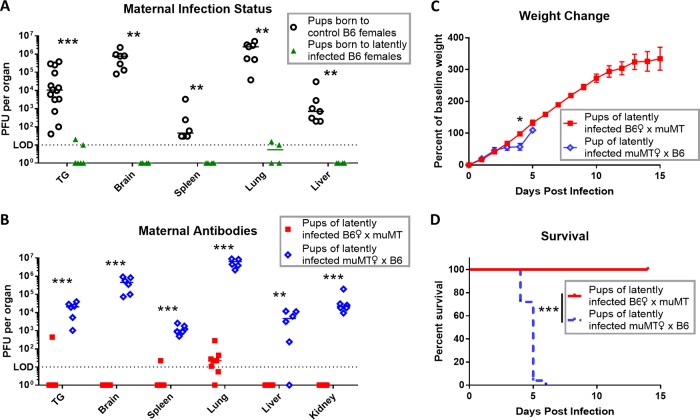
Maternal antibody protects pups from HSV-1 intranasal infection and disseminated disease. P0 to P2 pups were infected with 1 × 10^5^ PFU HSV-1 via the nasal route. After 3 dpi, pups were perfused, and titers in organs were determined. (A) Viral titers in organs from pups born to control naive or mock-infected B6 females (shown in black [*n =* 7]), compared with pups born to HSV-1 latently infected B6 females (shown in green [*n =* 4]). Breeding for pups used in panels B, C, and D was done as shown for Fig. 4E and F. (B) Viral titers in organs of pups born to latently infected B6 females (red [*n =* 8]) compared with pups born to latently infected muMT females (blue [*n =* 6]). (C and D) Weight change (C) and survival (D) of pups born to latently infected B6 females (red [*n =* 8]) or muMT females (blue [*n =* 25]) after neonatal intranasal infection with 1 × 10^4^ PFU HSV-1. Data are represented as individual animals and medians (A and B) or means with SEM (C). Statistical significance was determined by Mann-Whitney test (A and B), unpaired parametric *t* test (C), or log-rank test (D). *, *P* < 0.05; **, *P* < 0.005; ***, *P* < 0.001. Data represent 2 independent experiments.

Next, we addressed the hypothesis that HSV-specific maternal IgG could protect neonatal mice from HSV-1 infection. We used intranasal infection within 2 days postpartum to model perinatal acquisition of HSV. Compared to neonates from control (naive and mock-infected) B6 females, pups born to latently infected B6 females showed significantly reduced viral titers 3 dpi in all organs tested, including brain and TG (Fig. 6A). To test for the specific role of maternal antibodies, we infected pups from the mixed breeding scheme as described above (Fig. 4E and F). Pups from latently infected B6 females showed reduced titers in multiple tissues relative to pups from latently infected muMT females (Fig. 6B). While weight change was only significantly different at 5 dpi (Fig. 6C), pups from latently infected muMT females uniformly succumbed to infection by 6 dpi, while pups from latently infected B6 females all survived (Fig. 6D). Therefore, we concluded that maternal antibodies significantly protected neonates from disseminated viral infection and mortality.

### Maternal antibodies protect neonates from HSV-1 ocular infection and subsequent neurological spread from the TG.

**FIG 7:**
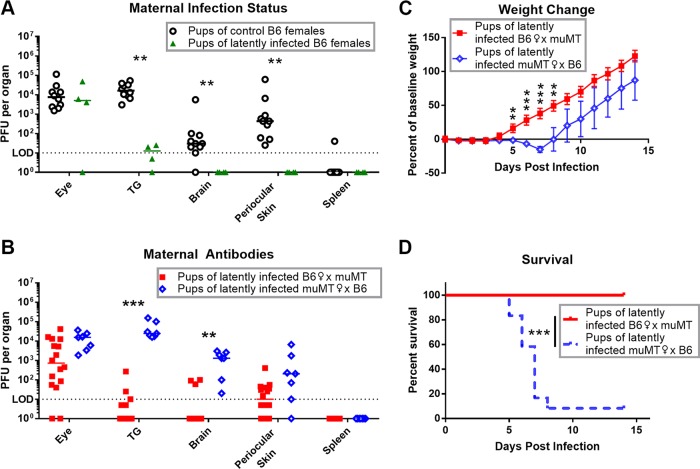
Maternal antibody protects pups from HSV-1 ocular infection and neurological spread. P14 to P16 pups were corneally infected with 1 × 10^5^ PFU HSV-1. (A) Viral titers in organs from pups born to control naive or mock-infected B6 females (black [*n =* 10]) compared with pups born to HSV-1 latently infected B6 females (green [*n =* 4]). Breeding for pups used in panels B, C, and D was done as shown for Fig. 4E and F. (B) Viral titers in organs 3 dpi of pups born to latently infected B6 females (red [*n =* 16]) compared with pups born to latently infected muMT females (blue [*n =* 7]). (C and D) Weight change (C) and survival (D) of pups born to latently infected B6 females (red [*n =* 14]) compared with pups born to latently infected muMT females (blue [*n =* 24]). Data are represented as individual animals and medians (A and B) or means with SEM (C). Statistical significance wsa determined by Mann-Whitney test (A and B), unpaired parametric *t* test (C), or log-rank test (D). **, *P* < 0.005; ***, *P* < 0.001. Data represent 2 to 3 independent experiments.

Following intranasal challenge, there are multiple routes of viral spread from the nasal passages into the central nervous system (CNS) and TG. To more specifically examine neurological spread via the TG, we reverted to the ocular infection model. Following primary infection of the cornea and retrograde transport to the TG, virus traffics via the nerves to the periocular skin and brain—often without systemic infection (27, 28). Older pups were necessary for these experiments because mice do not fully open their eyes before P12. While viral titers in the eye were similar, TG from pups born to latently infected B6 females supported significantly reduced viral burdens relative to pups born to uninfected control B6 females (Fig. 7A). Subsequently, there were reduced titers in the periocular skin and, importantly, reduced titers in the brain. We also compared HSV-1 infection in the pups of latently infected muMT or B6 females bred to naive males of the opposite genotype, as described above (Fig. 4E and F). Once again, there were no differences in viral titers in the eyes, but pups of latently infected B6 female mice showed reduced titers in their TG and brains relative to pups of latently infected muMT female mice (Fig. 7B). Furthermore, there was significant weight loss and mortality observed in pups born to latently infected muMT females relative to pups born to B6 females (Fig. 7C and D). Taken together, these data demonstrate that HSV-specific maternal IgG protects neonatal neural tissue from viral spread and subsequent morbidity and mortality.

## DISCUSSION

The estimated incidence of neonatal HSV infection is between 1 in 3,200 to 10,000 births, and reported rates are increasing (20, 21, 36–38). Even with antiviral intervention, neonatal HSV causes significant morbidity related to neurological infection (21). In this study, we show that maternal antibodies can transfer into fetal and neonatal neural tissue and have a significant impact on replication and neurological spread of HSV-1 in the newborn. This work validates a preventative strategy that may be broadly applicable to HSV and other fetal and neonatal neurological infections. Although previous work has supported the hypothesis that maternal immunity can protect against neonatal HSV sequelae (21, 24), our study is the first to experimentally address this hypothesis using a mouse model combined with analysis of human neural tissue. Moreover, we have demonstrated unexpected localization of maternal IgG in the nervous system and its therapeutic potential against infection. The standard therapy for neonatal herpes is intravenous acyclovir, which is effective but requires high index of clinical suspicion. Even high-dose or long-term antiviral treatment fails to prevent the neurological sequelae of HSV encephalitis (39, 40). Although caesarean section can reduce transmission of HSV in cases where there are clinical lesions, neonatal acquisition often results from subclinical maternal shedding (21). Our work highlights an alternative, prophylactic approach whereby either passive or active immunization of HSV-seronegative mothers before parturition could prevent and mitigate the sequelae of neonatal HSV.

Several vaccines have been developed against HSV but have failed in clinical trials due to their inability to prevent horizontal adult-to-adult transmission (41, 42). None of these subunit vaccines, however, have been used in trials examining prevention of vertical transmission. Other HSV vaccines, including live attenuated and replication-defective vectors, produce a greater diversity of antigen-specific antibodies compared to their subunit predecessors (43–47). Childhood immunizations with such live attenuated virus vaccines may stimulate immune responses in females that will ultimately protect against neonatal disease. However, the risk of using live vaccines in pregnancy is likely to be unacceptable at the regulatory level. It might be fruitful, therefore, to reconsider the use of polyvalent subunit, inactivated, or disabled infectious single-cycle (DISC) virus preparations in at-risk women for the protection of their babies from neonatal HSV (48, 49).

Maternal immunization may also be an effective strategy against other neurotropic pathogens that affect newborns. The emergence of the Zika virus as a cause of microcephaly in fetuses and neonates is of significant public health concern (34, 50). Recent work has shown that human neutralizing antibodies against Zika markedly reduce maternal, placental, and fetal infection in mice (51). Given the emerging knowledge that IgG can access fetal neural tissue (52), this suggests that the maternal IgG therapy may be directly clearing virus from the fetus, in addition to attenuating maternal amplification of the virus. Additionally, maternal IgG could potentially transfer through the milk as well as the placenta (24, 53, 54). We are currently investigating whether pre- and postnatal sources of maternal IgG differ in their localization to the neonatal nervous system.

How exactly IgG prevents viral replication in the nervous system remains unknown. Previous work with HSV suggests that antibody-mediated protection requires immune cells, especially lymphocytes (28, 55, 56). Therefore, immune cell recognition and control of viral replication by cytolytic or noncytolytic means are possible mechanisms (57). Regardless, this raises the intriguing prospect that nascent immune cells in the newborn are capable of mounting an effective antiviral defense in the presence of maternal antibodies against the virus. How this differs from the adult memory response remains to be investigated and possibly exploited for therapeutic purposes. It also seems plausible that TG IgG could impact neural function. HSV induces progressive oxidative damage to neurons (58, 59), and antiviral TG IgG may therefore preserve neuronal function and prevent neurological sequelae after HSV infection. Alternatively, a subset of sensory neurons express Fcγ receptors (FcγRs) that can bind to immune complexes and increase neuronal excitability, which may in turn potentiate pain signaling (60–62). TG IgG might therefore be involved in pain pathology, such as postherpetic or trigeminal neuralgia. It remains to be investigated whether IgG infiltrating the TG impacts neurological function.

The ability of these antibodies to prevent neurological HSV is dependent upon the propensity of circulating IgG to enter the naive TG. Our data show that systemic IgG can access the TG independently of local infection and inflammation. This result contrasts with a recent study suggesting that access of protective antiviral IgG into the dorsal root ganglia requires virus-specific CD4^+^ T cells to permeabilize the blood-nerve barrier (BNB) (33). Some key differences in study design that could account for this disparity include use of different viruses (HSV-1/HSV-2), different sites of infection (ocular/genital), and different immune induction strategies (infection with WT virus/vaccination with an attenuated virus and subsequent challenge). An important additional explanation for these contrasting observations may be the proximity of the TG to the meninges. The TG resides within Meckel’s cave (or cavum trigeminale), an outpouching of the arachnoid mater containing cerebrospinal fluid (CSF) (63). IgG can normally be found in the CSF and is elevated under neuroinflammatory conditions (64). Therefore, it is possible that IgG passes into the TG directly from the surrounding CSF. Additionally, the meningeal lymphatics (65), which converge at the base of the brain (66), may also serve as a conduit for circulating IgG into the TG. This unique anatomical juxtaposition may allow IgG access to the TG that bypasses the BNB and could be a useful portal for therapeutics into this critical site of HSV infection.

We have yet to address whether maternal IgG can effectively combat neonatal HSV-2. Although the incidence of genital disease from HSV-1 is rising, HSV-2 remains the major cause worldwide (35, 67–69). While HSV-1 and -2 share 83% amino acid sequence identity, a number of genes are functionally divergent (70–73), and HSV-2 is more neurovirulent (74, 75). Therefore, it will be valuable to assess the protective role of maternal antibodies against neonatal HSV-2 infection and the degree of protection provided by cross-reactivity. Another shortcoming of the HSV mouse model is functional loss of the viral FcγR. The HSV glycoprotein E and I complex binds to the Fc region of human IgG (76, 77), an important mechanism of viral immune evasion (78–80). However, the gE/gI IgG binding is species specific and functionally inactive in the murine model (81). Development of mouse-adapted HSV with restored Fc-binding activity or using humanized mouse models will help address whether maternal antibody protection is hindered by this immunomodulatory function.

The longstanding concept of immune privilege in the nervous system has likely discouraged studies of immunity in naive neural tissue. Our observation of antibodies accessing naive TG was almost overlooked but helps explain a decades-old finding that showed antibodies attenuating the neural spread of HSV-1 (28). This simple finding led us to investigate human fetal TG and reconsider the role of maternal antibodies in combating neonatal HSV infection. We showed that maternal antibodies not only access fetal and neonatal neural tissue but also protected the neonate from neurological infection and mortality. This study broadens our understanding of antibody access into neural tissue and underscores the heretofore unappreciated potential of maternal antibodies in directly combating neurological infections in the fetus and newborn.

## MATERIALS AND METHODS

### Cells and virus.

The HSV-1 strain used in this study was 17syn+ (82). Virus stock preparation and the plaque assay were performed using Vero cells (African green monkey kidney cells) as previously described (82–84).

### Mice and animal procedures.

All procedures were performed in accordance with federal and university policies. Six- to 10-week-old male and female adult mice were used. C57BL/6 (B6) and muMT (B6.129S2-Ighmtm1Cgn/J) mice were purchased from The Jackson Laboratory (Bar Harbor, ME) and bred in the barrier facility in the Center for Comparative Medicine and Research at the Geisel School of Medicine at Dartmouth. For adult and neonatal corneal infections, corneas were scarified with a 25-gauge needle and inoculated with indicated amounts of virus in a volume of 5 µl as previously described (83). For all experiments involving latently infected muMT mice, these mice and wild-type (WT) controls were treated with 2 mg/ml of acyclovir in the drinking water between 2 dpi and 21 dpi to promote survival beyond acute infection. For neonatal intranasal infections, P0 to P2 male and female pups were inoculated with virus in a volume of 5 µl under isoflurane anesthesia as previously described (85). For viral titers of organs, tissue was harvested after cardiac perfusion with at least 10 ml of cold phosphate-buffered saline (PBS) per animal. Periocular skin was harvested as 6-mm skin punches directly caudal to the eye, made with a trephine punch (Intregra Miltex). All tissues and organs were collected in 1.5-ml tubes containing ~100 µl of 1-mm-diameter glass beads and 1 ml of Dulbecco’s modified Eagle’s medium (DMEM) containing 1% fetal bovine serum (FBS), 1% penicillin-streptomycin, and 1% amphotericin B. Sample homogenates were prepared by mechanical disruption in Mini-Beadbeater-8 (BioSpec Products) and then sonicated. Titers of homogenates were determined by plaque assay on Vero cells. For serum preparation, naive or latently infected mice were anesthetized with isoflurane, and the mandibular vein was punctured with a 5-mm lancet (Medipoint). Bleeds were collected into Eppendorf tubes and allowed to clot by stasis for 15 to 30 min at room temperature. The clotted blood was then spun down at 2,000 × *g* for 10 min at 4°C, and the serum supernatant was collected and stored at −80°C.

### Human autopsy samples.

Fresh human autopsy samples were dissected and immediately stored at −80°C. These samples were processed for biochemistry or cryosectioned for microscopy. To extract protein and nucleotides, the frozen tissue was ground to a fine powder in liquid nitrogen using a sterile mortar and pestle. The tissue was divided and further processed for protein and nucleotides separately. Protein was prepared by homogenization with a tissue blender (Omni International) in PBS plus a cOmplete protease inhibitor cocktail tablet (Roche). Crude protein extract was spun at 14,000 × *g* at 4°C for 10 min, and the supernatant was used. Nucleotides were extracted with the TRIzol reagent and protocol (Invitrogen) and stored at −80°C. To prepare for microscopy, frozen tissue was fixed in 4% formaldehyde postcryosection.

### Immunofluorescence.

From PBS-perfused mice, tissue was fixed with 4% formaldehyde by perfusion or *ex vivo* and cryopreserved in 30% sucrose in PBS. Ten-micrometer sections were prepared using a Leica CM1860 UV cryostat at −22 to 25°C. Tissue was mounted onto Colorfrost Plus microscope slides (Fisher Scientific). Sections were blocked with 5% normal goat serum (NGS) or 0.1% fish skin gelatin (FSG) and 0.1% Triton X-100 in PBS. Primary antibodies were incubated overnight at 4°C, and secondary antibodies were incubated for 1 to 2 h at room temperature in the dark. The antibodies and dilutions used were Alexa Fluor 488-conjugated F(ab′)_2_ fragment of goat anti-mouse (H+L) at 1:250 (Life Technologies, Inc.), Alexa Fluor 488-conjugated goat anti-mouse (H+L) at 1:250 (Life Technologies, Inc.), Alexa Fluor 488-conjugated AffiniPure goat anti-human IgG (H+L) at 1:250 (Jackson), phycoerythrin (PE)-conjugated rat anti-mouse CD45 (30-F11) at 1:100 (BD Pharmingen), PE-conjugated anti-mouse CD4 (L3T4) at 1:200 (EBioscience), Alexa Fluor 594-conjugated rat anti-mouse CD31 (MEC13.3) at 1:200 (BioLegend), and Alexa Fluor 555-conjugated goat anti-rabbit (H+L) at 1:250 (Life Technologies, Inc.). Microscopy images were acquired on an Axio Observer Z1 microscope with a motorized stage (Zeiss) with an EC Plan-Neofluar 10×/0.3 objective (Zeiss), and tiled images were stitched and processed using Zen Blue (Zeiss).

### Western blotting.

Samples were denatured in 50 mM Tris-HCl (pH 6.8) (Invitrogen), 100 mM dithiothreitol (DTT) (Sigma), 2% SDS (Invitrogen), 0.1% bromophenol blue (Sigma), and 10% glycerol (National Diagnostics) at 95°C for 5 min. Samples were run on 10% SDS-PAGE gels, transferred to polyvinylidene difluoride (PVDF) membranes, and blocked with ~5% skim milk in PBS with 0.1% polysorbate 20 (PBST). Primary and secondary antibody incubations were for 18 h at 4°C and 1 to 2 h at room temperature, respectively. For the modified Western blot, murine and human TG homogenates were used as the primary antibody. TG homogenate was created from perfused tissue that was homogenized with a tissue blender (Omni International) in PBS plus protease inhibitor (Roche) as described above. Binding of TG IgG to the blot was visualized using an appropriate secondary antibody. Recombinant viral glycoproteins gH/L, gB, gC, and gD and polyclonal antibodies as positive controls were generously provided by Roselyn J. Eisenberg and Gary H. Cohen (University of Pennsylvania [30]). The antibodies and dilutions used were rabbit anti-mouse β-actin (BioLegend) at 1:1,000, goat anti-rabbit IgG (H+L) conjugated to horseradish peroxidase (HRP) (BioRad) at ~1:35,000, goat anti-mouse IgG (H+L) conjugated to HRP (Biorad) at ~1:35,000, and goat anti-human IgG (H+L) conjugated to HRP (Thermo Fisher Scientific) at ~1:35,000.

### ELISA and BCA.

The enzyme-linked immunosorbent assay was performed using the EBioscience assay Mouse IgG Total ELISA Ready-SET-Go! The protein concentration was determined using the Pierce BCA assay kit (Thermo Fisher Scientific).

### ELISpot.

Perfused TG were digested in 200 U/ml of collagenase II (Gibco) for 1.5 h at 37°C and triturated with micropestles (Eppendorf). The ELISpot assay was performed with the Protein Detector AP ELISpot kit (KPL) with 10 µg/ml of goat anti-mouse IgG (KPL) for coating and 1 µg/ml of biotin-SP AffiniPure goat anti-mouse IgG (H+L) (Jackson) for detection.

### RT-PCR.

Real-time RT-PCR was used to measure IgG mRNA expression in mouse tissues. This was performed with primers and probes designed against an IgG1 heavy chain target sequence (GenBank accession no. AF542525) as previously described (86–88) (see Text S1 in the supplemental material). Briefly, total RNA was isolated from homogenized tissue samples using TRIzol RNA isolation reagents (Life Technologies, Inc.); total RNA (50 ng/µl) was then reverse transcribed using random hexamer primers with the qScript cDNA SuperMix (Quanta Biosciences). Finally, cDNA was used as a template for the real-time RT-PCR analysis based on the 5′ nuclease assay. Gene expression was normalized to the housekeeping gene coding for glyceraldehyde-3-phosphate-dehydrogenase (GAPDH). Relative quantification of IgG1 mRNA was calculated by the threshold cycle (ΔΔ*C*_*T*_) method.

10.1128/mBio.00678-17.1 TEXT S1  Supplemental methods. Download TEXT S1, DOCX file, 0.01 MB. Copyright © 2017 Jiang et al. 2017 Jiang et al. This content is distributed under the terms of the Creative Commons Attribution 4.0 International license.
