# Supplementary figures and images for: Maternal Antiviral Immunoglobulin Accumulates in Neural Tissue of Neonates To Prevent HSV Neurological Disease
Source: mBio. 2017 Jul 5;8(4):e00678-17. doi: 10.1128/mBio.00678-17 (PMC5573671; doi:10.1128/mBio.00678-17)

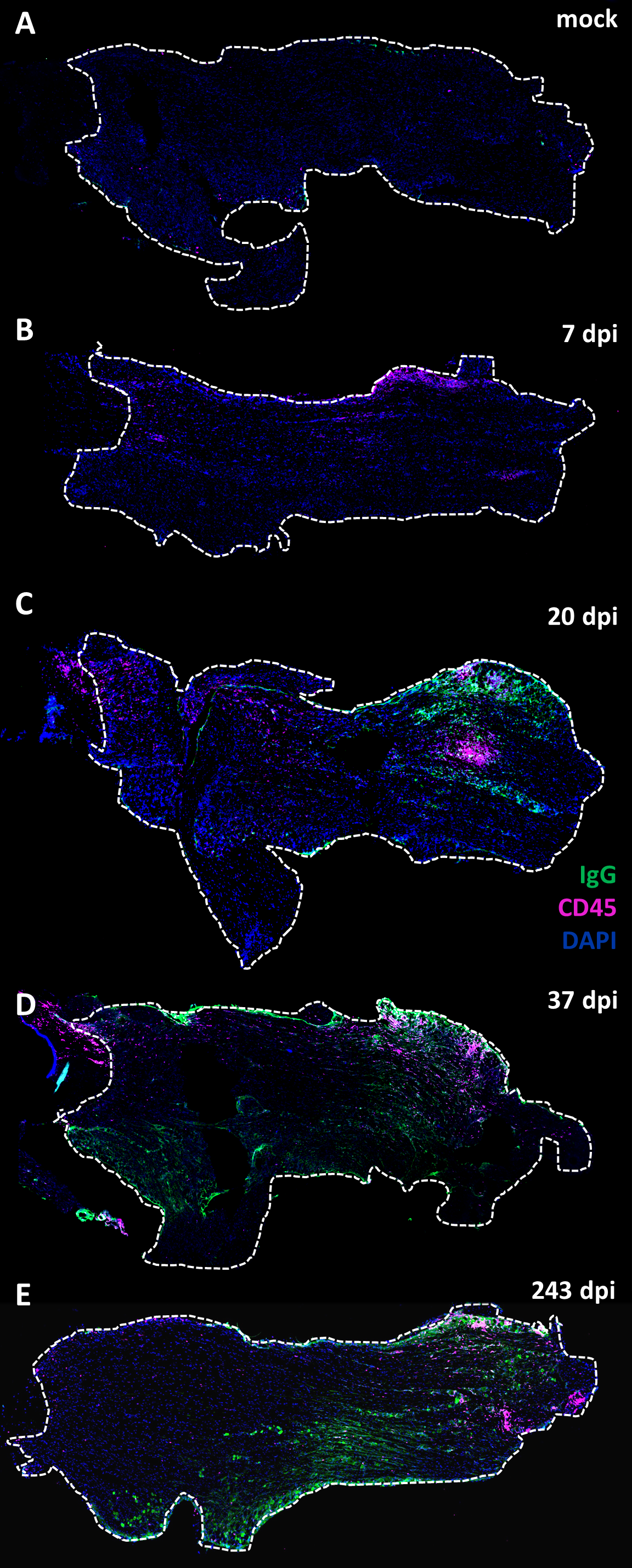

Supplement: FIG S1 [file mbo003173373sf1.tif]

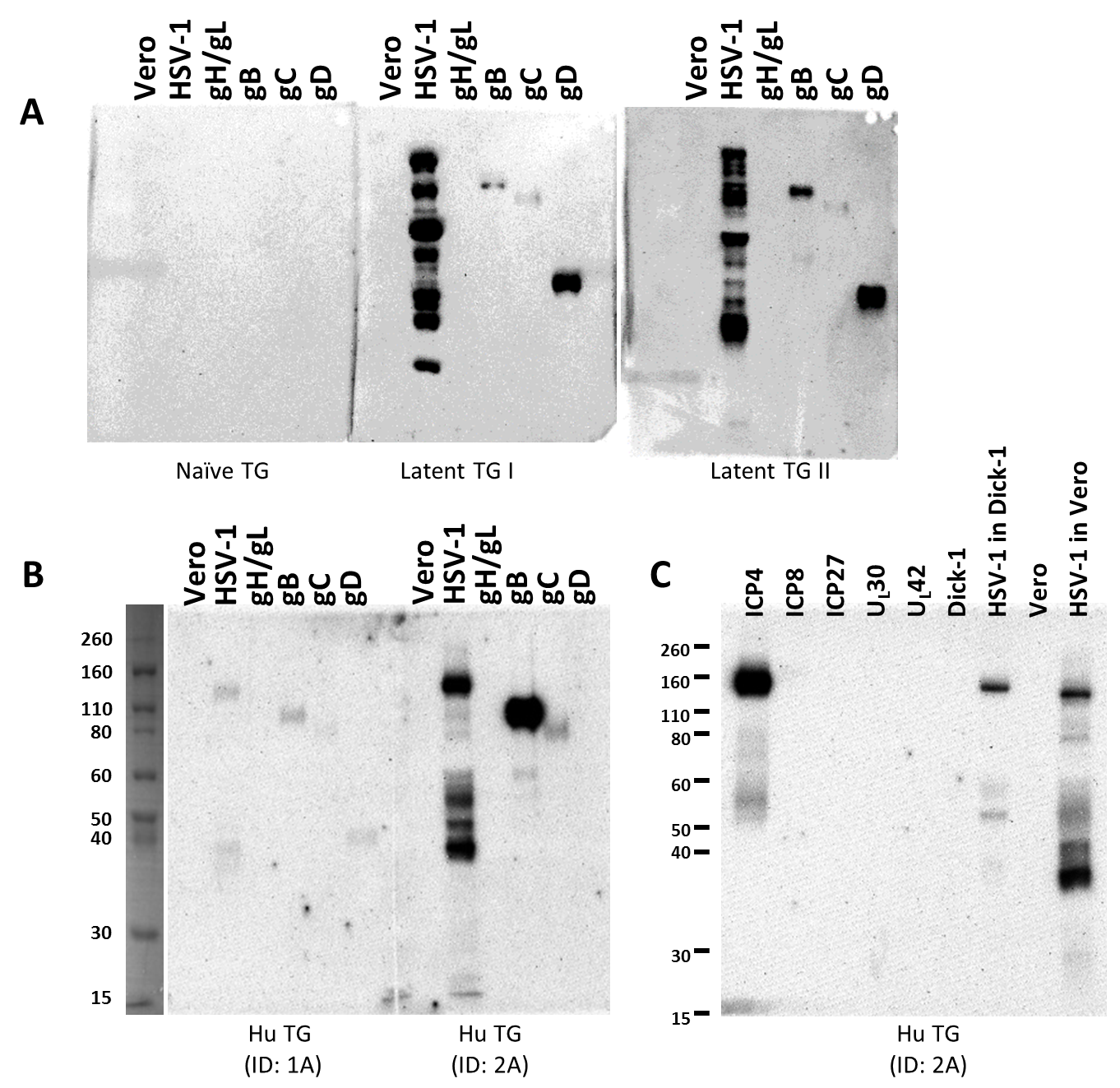

Supplement: FIG S2 [file mbo003173373sf2.tif]

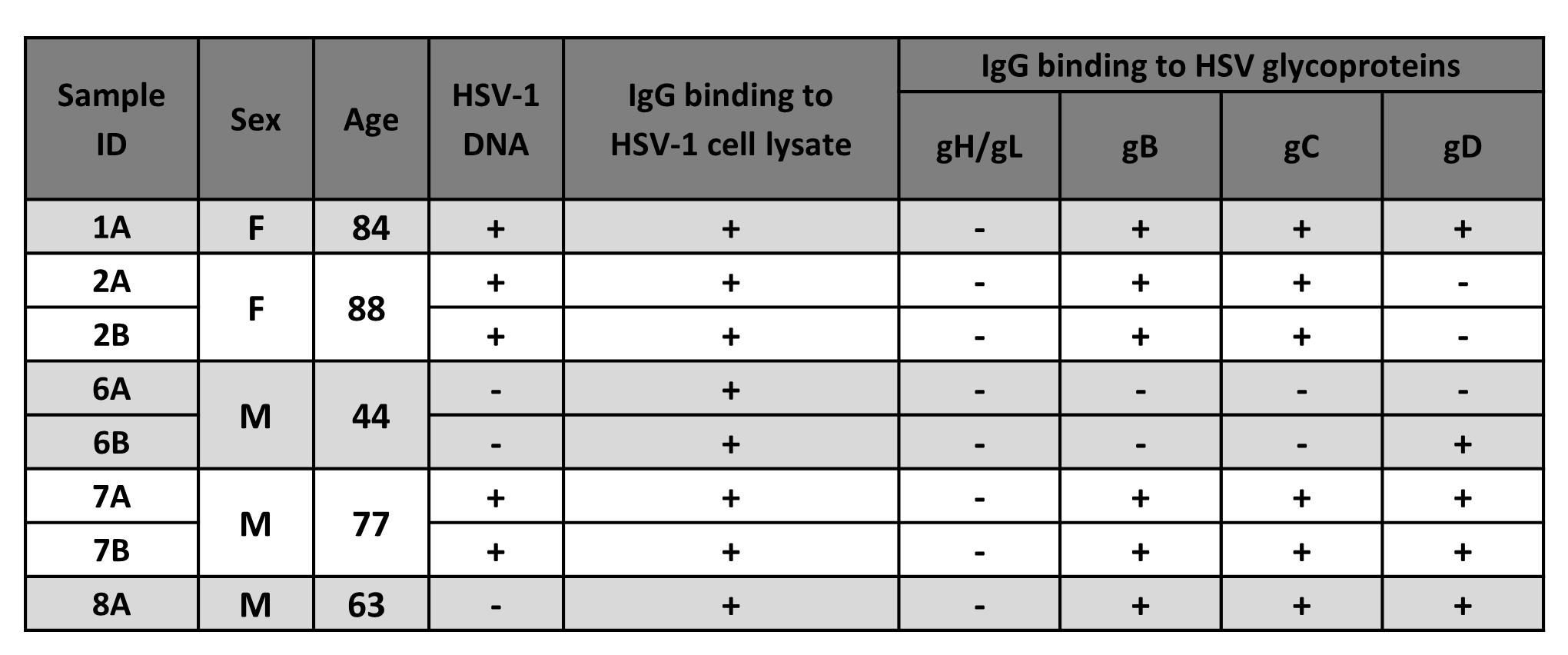

Supplement: TABLE S1 [file mbo003173373st1.tif]

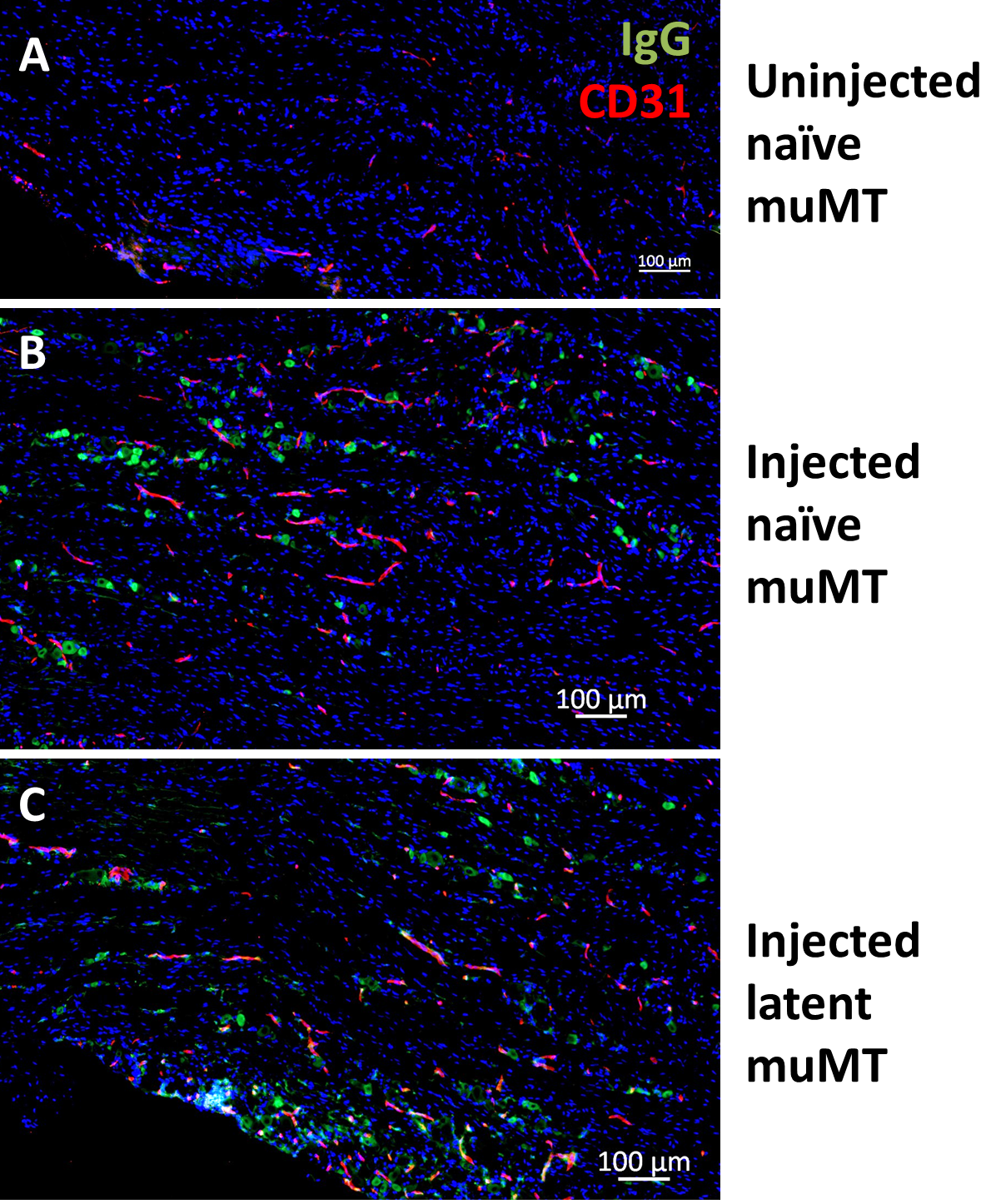

Supplement: FIG S3 [file mbo003173373sf3.tif]

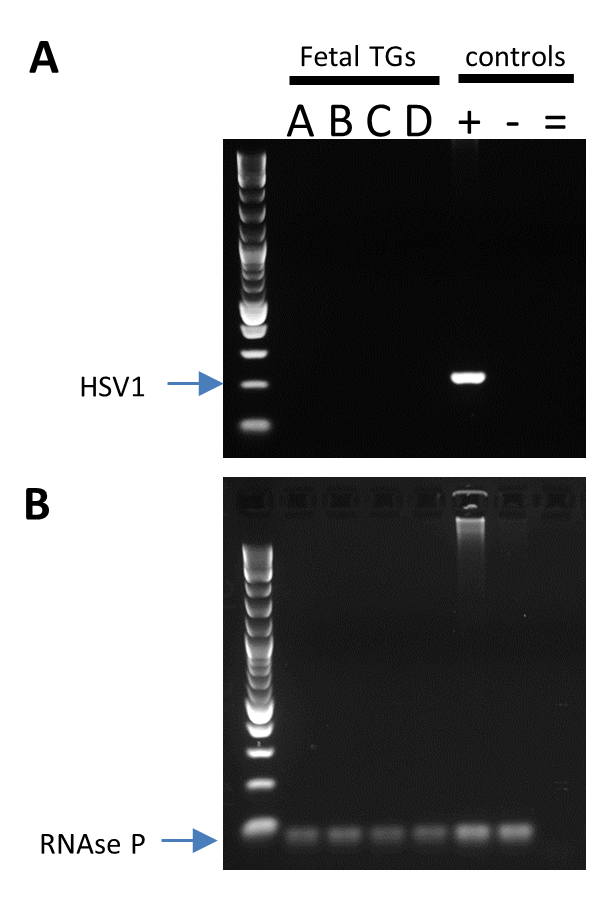

Supplement: FIG S4 [file mbo003173373sf4.tif]
