# Supplementary material for: Maternal Antiviral Immunoglobulin Accumulates in Neural Tissue of Neonates To Prevent HSV Neurological Disease
Source: mBio. 2017 Jul 5;8(4):e00678-17. doi: 10.1128/mBio.00678-17 (PMC5573671; doi:10.1128/mBio.00678-17)
Supplement: TEXT S1 [file mbo003173373s1.docx]

**Supplemental Methods**

***Western blot (continued).*** For supplemental modified Western blots, additional lysates were used. Purified ICP4 was generously provided by Dr. Neal DeLuca (University of Pittsburgh). Purified ICP8 and ICP27 were generously provided by Dr. David Knipe (Harvard Medical School). Purified U_L_30 and U_L_42 were generously provided by Dr. Donald Coen (Harvard Medical School). HSV-1 infected Dick-1 cells (immortalized human foreskin fibroblasts) were also used as lysate.

***PCR.*** GoTaq® green buffer and polymerase (Promega) and the following primer sets were used: HSV-1 gD forward 5’-ATCACGGTAGCCCGGCCGTGTGACA-3’ and reverse 5’-CATACCGGAACGCACCACAC-3’ (annealing 68°C)(87); RNAse P forward 5’-CCAAGTGTGAGGGCTGAAAAG-3’ and reverse 5’-TGTTGTGGCTGATGAACTATAAAAGG-3’(annealing 60°C)(88).
